# Supplementary material for: The genome sequence of a long-legged fly, Scellus notatus (Fabricius, 1781)
Source: Wellcome Open Res. 2025 Feb 28;10:117. [Version 1] doi: 10.12688/wellcomeopenres.23764.1 (PMC11914874; doi:10.12688/wellcomeopenres.23764.1)
Supplement: Peer review history

# Reviewer report: Reviewer response for version 1

**DOI:** 10.21956/wellcomeopenres.26212.r119825

**Published:** 2025-03-17

**Authors:** Henrique Antoniolli

**Affiliations:** 1 Universidade Federal do Rio Grande do Sul (UFRGS), Porto Alegre, Brazil

The present study reports a highly contiguous draft genome assembly of *Scellus notatus*. Even though the background section is short, it concisely describes the species and what is known about its biology, including the rationale for sequencing its genome. Methods and protocols are well described and referenced. The produced data are deposited in public repositories, which accession codes are clearly stated throughout the text. Even though genes were neither predicted nor annotated, all statistics about the genome assembly yielded satisfactory results. Therefore, I recommend indexing this paper.

Are sufficient details of methods and materials provided to allow replication by others?

Yes

Is the rationale for creating the dataset(s) clearly described?

Yes

Are the datasets clearly presented in a useable and accessible format?

Yes

Are the protocols appropriate and is the work technically sound?

Yes

Reviewer Expertise:

Genomics, Evolution

I confirm that I have read this submission and believe that I have an appropriate level of expertise to confirm that it is of an acceptable scientific standard.

# Reviewer report: Reviewer response for version 1

**DOI:** 10.21956/wellcomeopenres.26212.r119827

**Published:** 2025-03-12

**Authors:** Komal Kumar Raja

**Affiliations:** 1 Baylor College of Medicine, Houston, TX, USA

The article by Falk S. *et al.* presents the genome sequence of long-legged fly, *Scelus* *notatus*. The authors have briefly introduced the ecological and morphological aspects of this species. The genome sequence has been generated using standard methods and informatics tools. 59x coverage is commendable and the percentage of sequences (99.19%) aligned to chromosomal scaffolds is impressive. The quality metrics such as QV, BUSCO and k-mer completeness meet the expected benchmarks suggesting that the assembly is high quality. The methodology is robust.

 1. Can the authors discuss briefly why the k-mer completeness of individual haplotypes is low compared to the assembly? Are the gaps due to repetitive sequences?

 2. Please be consistent between Hi-C and HiC in the manuscript.

 Overall, this is robust data and a good resource.

Are sufficient details of methods and materials provided to allow replication by others?

Yes

Is the rationale for creating the dataset(s) clearly described?

Yes

Are the datasets clearly presented in a useable and accessible format?

Yes

Are the protocols appropriate and is the work technically sound?

Yes

Reviewer Expertise:

Single cell genomics, bioinformatics, genomics

I confirm that I have read this submission and believe that I have an appropriate level of expertise to confirm that it is of an acceptable scientific standard.
